# *Klebsiella* LPS O1-antigen prevents complement-mediated killing by inhibiting C9 polymerization

**DOI:** 10.1038/s41598-024-71487-z

**Published:** 2024-09-05

**Authors:** Frerich M. Masson, Salvör Káradóttir, Sjors P. A. van der Lans, Dennis J. Doorduijn, Carla J. C. de Haas, Suzan H. M. Rooijakkers, Bart W. Bardoel

**Affiliations:** Medical Microbiology, University Medical Center Utrecht, Utrecht, The Netherlands

**Keywords:** Immunology, Microbiology

## Abstract

The Gram-negative bacterium *Klebsiella pneumoniae* is an important human pathogen. Its treatment has been complicated by the emergence of multi-drug resistant strains. The human complement system is an important part of our innate immune response that can directly kill Gram-negative bacteria by assembling membrane attack complex (MAC) pores into the bacterial outer membrane. To resist this attack, Gram-negative bacteria can modify their lipopolysaccharide (LPS). Especially the decoration of the LPS outer core with the O-antigen polysaccharide has been linked to increased bacterial survival in serum, but not studied in detail. In this study, we characterized various clinical *Klebsiella pneumoniae* isolates and show that expression of the LPS O1-antigen correlates with resistance to complement-mediated killing. Mechanistic data reveal that the O1-antigen does not inhibit C3b deposition and C5 conversion. In contrast, we see more efficient formation of C5a, and deposition of C6 and C9 when an O-antigen is present. Further downstream analyses revealed that the O1-antigen prevents correct insertion and polymerization of the final MAC component C9 into the bacterial membrane. Altogether, we show that the LPS O1-antigen is a key determining factor for complement resistance by *K. pneumoniae* and provide insights into the molecular basis of O1-mediated MAC evasion.

## Introduction

*Klebsiella pneumoniae* is a Gram-negative bacterium that has gained global attention in the past decades for its association with infection-related deaths and increasing resistance to antibiotics^1^. Next to resisting antibiotics, multiple *Klebsiella* serotypes have been reported to withstand bacterial killing by human immune proteins in serum^2^, but the underlying mechanistics remained understudied.

Killing of Gram-negative bacteria in human serum is mediated by the complement system, a conserved part of human immunity. It consists of soluble plasma proteins that can form a proteolytic cascade to ultimately generate so-called membrane attack complexes (MAC pores). These ring-shaped complexes insert in the outer membrane of Gram-negative bacteria and efficiently kill them^3^. Activation of the complement system can happen via three distinct pathways: the so-called classical pathway (CP) is engaged after binding of antibodies to surface-bound antigens, the lectin pathway (LP) that recognizes foreign sugar patterns, and finally the alternative pathway (AP) which functions to amplify the CP and LP but is also continuously active at low levels^4^. All three pathways converge into the cleavage of the central protein C3 and covalent binding of its activated product C3b to the target surface^4^. Here, unless negatively regulated, deposited C3b molecules can further amplify C3 cleavage via formation of AP C3 convertases that effectively opsonize bacteria with large amounts of C3b. Upon high enough C3b densities, a yet unknown mechanism leads to a change in substrate specificity towards C5 and then form the C5 convertase. This C5 convertase marks the beginning of the terminal part of complement activation. The C5 convertase will cleave C5 into C5b and the smaller chemoattractant C5a, which attracts neutrophils to kill *Klebsiella*. The unstable C5b is then immediately bound by C6, to form the more stable C5b6 complex^5,6^. The ensuing binding of C7 stably anchors the MAC precursor in the bacterial membrane^7^. Binding of C8 to the membrane-bound C5b-7 complex will lead to structural changes in C8, which will insert a transmembrane domain into the bacterial membrane^8,9^. Finally, up to 18 copies of C9 will bind to the C5b-8 complex in a step-wise manner and form a ring-shaped pore complex that pierces through the bacterial outer membrane, ultimately resulting in bacterial cell death ^3,10,11^.

Even though the MAC can efficiently kill serum-sensitive Gram-negative bacteria, many bacteria can withstand killing via the MAC^12,13^. Gram-positives are inherently resistant to killing via MAC pores ^14^ because their thick peptidoglycan layer shields the bacterial membrane. While Gram-negatives are naturally sensitive to the MAC, many clinically important Gram-negative bacteria have evolved resistance mechanisms to withstand killing in human serum. *Klebsiella pneumoniae* expresses a variety of polysaccharide surface structures to cover its cell envelope as a protective barrier. Namely, capsule (K-antigen), LPS (O-antigen), and exopolysaccharides (EPS). To date, 134 K-types and up to 11 distinct O-antigens have been identified^15,16^. The EPS is associated with the formation of bacterial biofilm, but there is still little information available regarding this virulence factor^17^. Previous reports correlate the presence and length of O-antigen with survival in serum for *Klebsiella* and other Gram-negatives^18–22^. Of note, serotypes O1 and O2 generally account for more than half of all infection-related *K. pneumoniae* isolates^23,24^. For *Klebsiella*, one polysaccharide structure that has been reported to contribute to survival in serum, is the LPS O-antigen ^19,20,25,26^. The O-antigen consists of repeating units of varying length of sugar moieties that are covalently bound to the LPS core^27^. The two most common serotypes among drug-resistant *K. pneumoniae* are O1 and O2^23,24^. Both O1 and O2 serotypes have repeating units of d-Galactan I linked to the LPS core^27^. However, for O1-serotypes, these d-Galactan I units are covered with additional repeating units of d-Galactan II (here called O1-cap)^28^. Although the presence of the O-antigen polysaccharide has been described to contribute to serum survival ^29^, the exact mechanism by which it blocks complement-mediated killing remains elusive.

In this paper, we show that expression of LPS O1-antigen is associated with increased survival in serum. This resistance is not caused by blocking complement activation as such. Rather, C3b deposition and C5 conversion are increased, preventing correct insertion and polymerization of C9 into a functional MAC pore in the bacterial outer membrane. Incorrectly formed MAC pre-pores are then released in their soluble form from the bacterial surface. This study therefore gives insights into how *Klebsiella* LPS O1-antigen enables bacteria to evade MAC-mediated killing.

## Results

### Expression of LPS O1-antigen correlates with resistance to complement-mediated killing

**Fig. 1:**
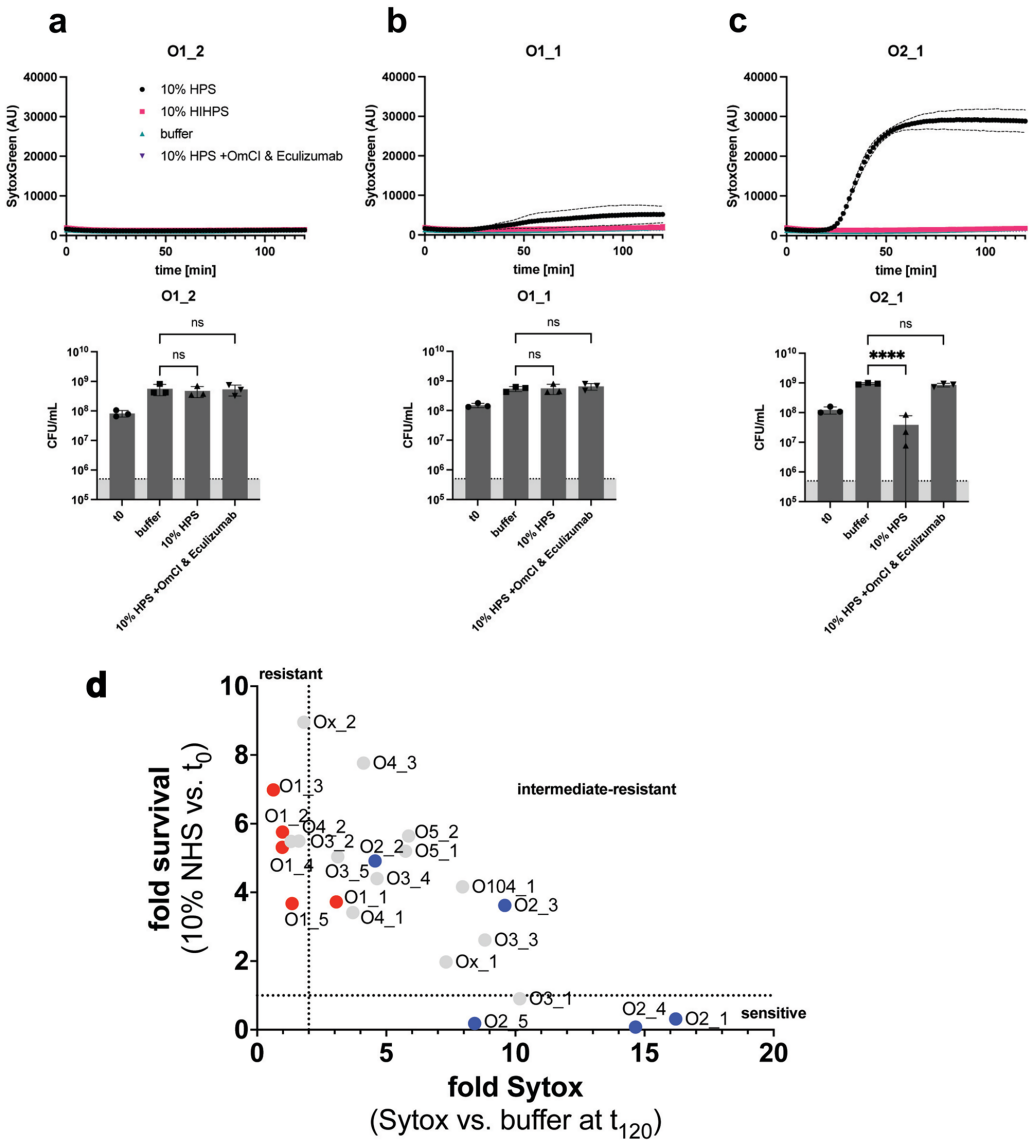
Characterization of serum resistance of clinical *K. pneumoniae* isolates. Bacteria were exposed to 10% NHS, 10% HI NHS or 10% NHS with added C5 inhibitors at 37 °C for 120 min. Inner membrane damage was assessed using SytoxGreen DNA stain. After serum exposure, serial dilutions of bacteria in PBS were plated and CFUs were assessed the next day. The grey bar is indicating the detection limit of the assay. (**a**) Serum-resistant phenotype showing no Sytox signal and no decrease in CFU counts. (**b**) Intermediate-resistant phenotype with minor Sytox signal but no decrease in CFU after serum exposure. (**c**) Serum-sensitive phenotype with high Sytox signal and decrease in CFU. (**d**) Overview of Sytox value and survival of all tested strains. Fold survival was calculated by dividing CFUs at t = 0 by CFUs after exposure to 10% NHS. Strains carrying an LPS O1-antigen are marked with red dots, those carrying the O2a-antigen are marked in blue. Dotted lines represent respective cut-off values for classification of resistance. Data shown represent mean value ± SD of three independent experiments. In (**d**) only mean values are shown. Statistical analysis was done using a paired one-way ANOVA with Tukey’s multiple comparisons’ test. Significance shown as ns, non-significant, or ****p ≤ 0.0001.

To understand complement resistance in *Klebsiella pneumoniae*, we first screened a panel of 23 clinical isolates, expressing a variety of different capsular and O-antigen serotypes. Bacteria were sequenced, and O- and K-type were assessed using the KAPTIVE online tool^30,31^. For these strains, we determined the capacity to survive exposure to 10% normal human serum (NHS). For this, we used a membrane non-permeable DNA dye (Sytox) as a marker of membrane integrity. Upon membrane damage (e.g. via MAC deposition), Sytox can flow into the bacterial cell and becomes fluorescent after binding to DNA^32^. In addition, bacterial survival was analysed by colony enumeration. Strains were classified in three categories: resistant, intermediate-resistant and sensitive. Strains were classified as ‘resistant’ if they survived serum exposure (Fig. 1a lower panel) and had no more than a twofold above background increase in Sytox signal (Fig. 1a upper panel). ‘Intermediate-resistant’ were strains that survived serum exposure an showed a more than twofold increase in membrane damage over background (Fig. 1b). The remaining strains with a high membrane damage and decrease in CFU, were termed as ‘sensitive’ (Fig. 1c). Based on this analysis, 7 out of 23 clinical *K. pneumoniae* isolates were serum resistant, 12 strains were determined to be intermediate-resistant and four strains were serum-sensitive (Fig. 1d). To determine if killing was MAC-dependent, we added two well-described C5 cleavage inhibitors (OmCI and Eculizumab^33,34^). For all intermediate-resistant and serum-sensitive strains, membrane damage and killing were inhibited in the presence of C5 inhibitors (Figs. S1, S2), indicating that the observed killing was MAC-dependent.

To show the correlation for multiple strains between Sytox and CFU, we plotted bacterial survival against relative Sytox over background after 2 h of serum exposure (Fig. 1d). In general, high Sytox values correlated with sensitivity to serum-mediated killing. We analyzed the clinical isolates for possible common determinants of serum resistance. A correlation between O-antigen and serum survival has been reported by others for various Gram-negative bacteria and *Klebsiella *^18,25^. In *Klebsiella*, the O1 serotype is associated with serum resistance, while the O2 serotype is much more sensitive^25,35,36^. In our study, all strains presenting an LPS O1-antigen grouped together as resistant or intermediate-resistant to serum exposure, while strains bearing only an O2-antigen were either fully sensitive or intermediate-resistant (Fig. 1d). All other classes of O-antigen present in our panel of strains showed not such a clear distinction. Because of the clear link between O1-antigen expression and serum resistance, we set out to further elucidate the role of the O1-antigen on MAC resistance.

### Deletion of LPS O1-antigen renders serum resistant strain fully sensitive to MAC

**Fig. 2:**
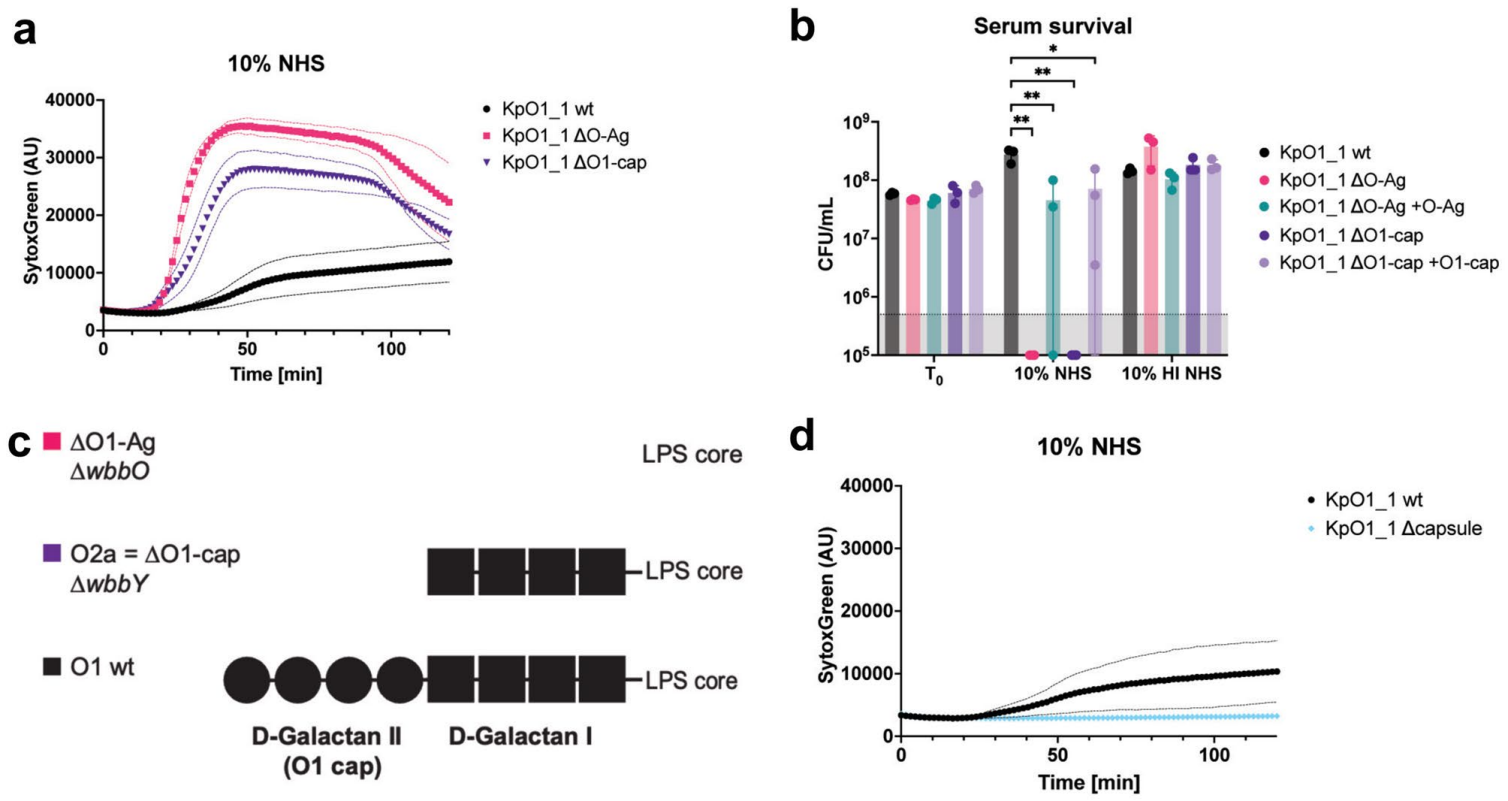
Deletion of O1-antigen or O1-antigen cap, but not capsule, render resistant strain fully sensitive to serum killing. (**a**) Bacteria were exposed to 10% NHS at 37 °C for 120 min. Inner membrane damage was assessed using SytoxGreen DNA stain and measured every 90 s in a multi-well plate reader assay. (**b**) Bacteria were exposed to 10% NHS or 10% HI NHS 37 °C for two hours. After serum exposure, serial dilutions of bacteria in PBS were plated, the next day CFU counts were assessed and colony forming units per ml were calculated. The grey bar is indicating the detection limit of the assay. (**c**) Schematic overview of the O1 and O2a antigen, and the knockout mutants used in this study. (**d**) Bacteria were exposed to 10% NHS at 37 °C for 120 min. Inner membrane damage was assessed using SytoxGreen DNA stain and measured every 90 s in a multi-well plate reader assay. (**a**,**b**,**d**) Data represent mean values ± SD (thin lines and error bars, respectively) of three independent experiments. Statistical analysis was done using a paired one-way ANOVA with Tukey’s multiple comparisons’ test. Significance shown as *p ≤ 0.05 or **p ≤ 0.01.

To more definitely show the involvement of the O1 antigen in serum resistance, we deleted specific genes involved in O1 synthesis. To delete the entire O1 antigen (ΔO-Ag), we created a knockout mutant lacking the *wbbO* gene, an early gene in O-antigen synthesis, resulting in a total loss of O-antigen expression^26,37^. We validated the mutants by antibody staining with an anti-O1 monoclonal antibody using flow cytometry (Fig. S3). We then exposed the strains to NHS to check for MAC sensitivity. Deletion of *wbbO* (ΔO-Ag) led to an increase in inner membrane permeability in response to serum, as determined by increase in Sytox (Fig. 2a). Furthermore, we observed total loss of viability upon serum exposure, in contrast to the wild type (Fig. 2b).

To assess ift the complete O1-antigen is required for serum resistance, we created another deletion mutant without the O1-cap. The genes *wbbY* and *wbbZ* are necessary for the synthesis of the d-galactan II repeating unit on top of d-galactan I, specific for the O1-antigen ^16,38,39^. To specifically delete the O1 cap (ΔO1-cap), we created a mutant of the KpO1_1 strain lacking the *wbbY* gene, which is sufficient to prevent O1 cap synthesis (Fig. 2c). Deletion of the O1-cap led to an increase in inner membrane permeability (Fig. 2a) and loss of viabiolity (Fig. 2b) upon serum exposure compared to wild-type. Complementation of the knockout strains with a plasmid containing the respective genes lead to reversion of the observed susceptibility to serum-dependent killing (Fig. 2b). This data indicates that the full O1-antigen comprising both O2a-antigen and O1-antigen cap is required for serum-resistance in *K.* *pneumoniae*.

To confirm that the O1-antigen is also causing serum resistance in other LPS O1-antigen-bearing strains, we set out to create knockouts of the genes *wbbO* and *wbbY* in another *K. pneumoniae* isolate (KpO1_2). The previously fully serum-resistant strain KpO1_2 became sensitive to MAC-induced inner membrane damage through deletions of either *wbbO* or *wbbY* (Fig. S4). Besides the O-antigen, the capsule has been described to have a role in serum resistance^40^. In contrast to the O1-antigen, deletion of capsule (Δ*wbaP)*^41,42^ in strain KpO1_1 had no effect on serum survival (Fig. S5) and did not lead to an increased membrane damage upon serum exposure (Fig. 2d).

To further validate the importance of the O1-cap on MAC-resistance, we wondered if we could make an O2a-antigen bearing strain resistant by overexpressing the O1 cap. Therefore, we introduced a plasmid carrying the genes *wbbY* and *wbbZ* into the serum-susceptible strain KpO2_1, introducing the O1-cap and changing the O-antigen from O2a to O1 (Fig. S6a). Upon expression of these two additional genes, the previously serum-sensitive strain became serum-resistant, as determined by both reduction in Sytox signal (Fig. S6b) and survival in 10% NHS (Fig. S6c,d). In conclusion, we show that the O1-antigen determines serum sensitivity in two O1-expressing *K. pneumoniae* strains.

### O1-antigen does not block early stages of complement activation

**Fig. 3:**
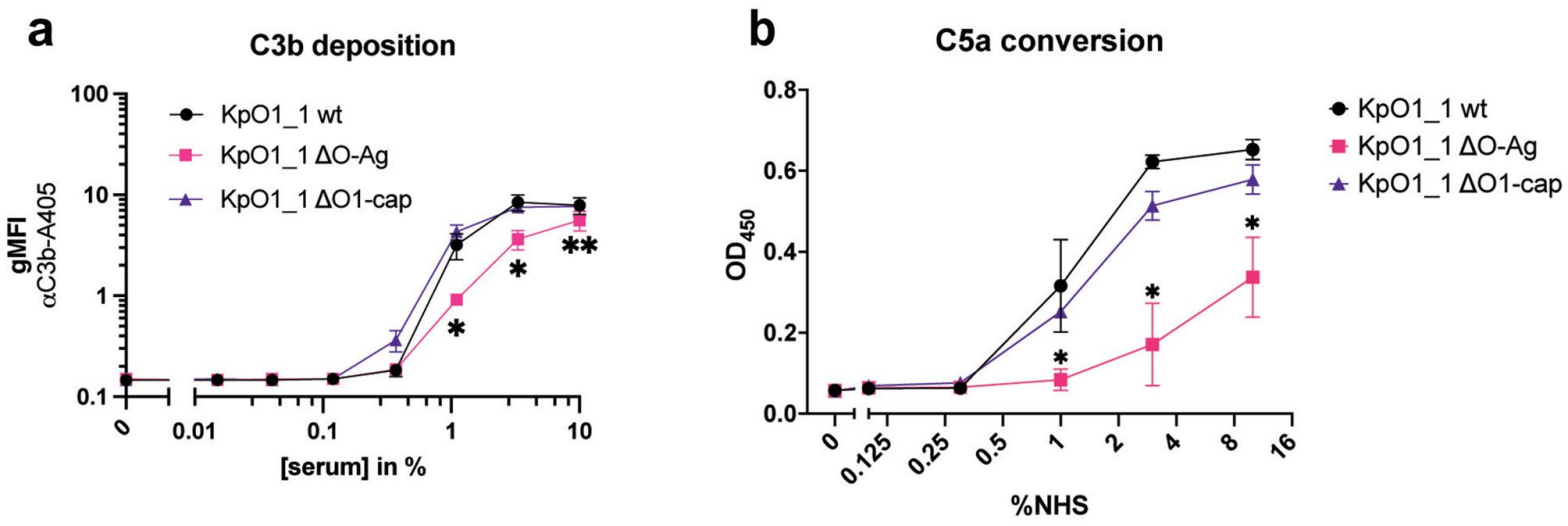
O-antigen does not block early complement activation. (**a**) Bacteria were incubated with serial dilutions of normal human serum (NHS) which was supplemented with C5-inhibitors Eculizumab (15 µg/ml) and OmCI (6 µg/ml) at 10% serum. After washing, C3b deposition was determined through flow cytometry by measuring geometric mean fluorescence of α-C3b-A405 antibody. (**b**) Bacteria were exposed to NHS at 37 °C for 15 min. Supernatant was collected, and C5a conversion was measured with an ELISA assay. Data shown in both panels represent mean values ± SD of three independent experiments. Statistical analysis was done using paired t-test. Significance shown as *p ≤ 0.05 or **p ≤ 0.01.

The clear effect of the O1-antigen on MAC-mediated killing raised the question at what stage the O1 antigen interferes with the complement cascade. Antibody deposition of both IgG and IgM from serum showed no significant differences between KpO1_1 and the cap knock-out (Fig. S7a,b). To check if the O1-antigen does not completely block complement activation, we first checked for C3b deposition. This activation product covalently binds to the bacterial surface and is the culminating point of all pathways of early complement activation leading to opsonization^4^. C3b deposition was analyzed with a fluorescently labelled C3b specific antibody using flow cytometry. Both the KpO1_1 wildtype and O1-cap mutant deposited C3b to comparable levels at 10% NHS (Fig. 3a). However, deletion of the entire O1-antigen containing the O1 cap and the O2a-antigen (ΔO-Ag) lead to a decrease in C3b deposition compared with the wild-type at lower serum concentrations. These results indicate that the effect of the O1-antigen on MAC-mediated killing is caused by a difference in the later stage of the complement cascade. We therefore set out to assess activation of further downstream complement components.

The cleavage of C5 into C5b and C5a marks the beginning of the terminal step of complement activation, the formation of a MAC pore. At high density, covalently bound C3b molecules form the basis of the C5 convertase^43,44^. To determine if the O-antigen poses a hindrance to complement activation at a later stage, we determined the C5a formation rates by the *K. pneumoniae* mutants. Serum-resistant Gram-negatives expressing an LPS O-antigen, can potently convert C5 into C5a and C5b^21^. We observed that the wild-type KpO1_1 strain and the O1 cap mutant showed potent C5 conversion upon exposure to serum. In contrast, the deletion of the complete O-antigen lead to a decrease in C5a conversion (Figs. 3b, S8a). This indicates that the presence of an O-antigen in *K. pneumoniae* can increase the conversion of C5 in human serum. However, how this increased C5 conversion influenced resistance to MAC, remained unclear. For this reason, we continued to investigate the influence of the O1-antigen on MAC formation.

### Presence of O1-antigen increases the deposition of terminal complement components

**Fig. 4:**
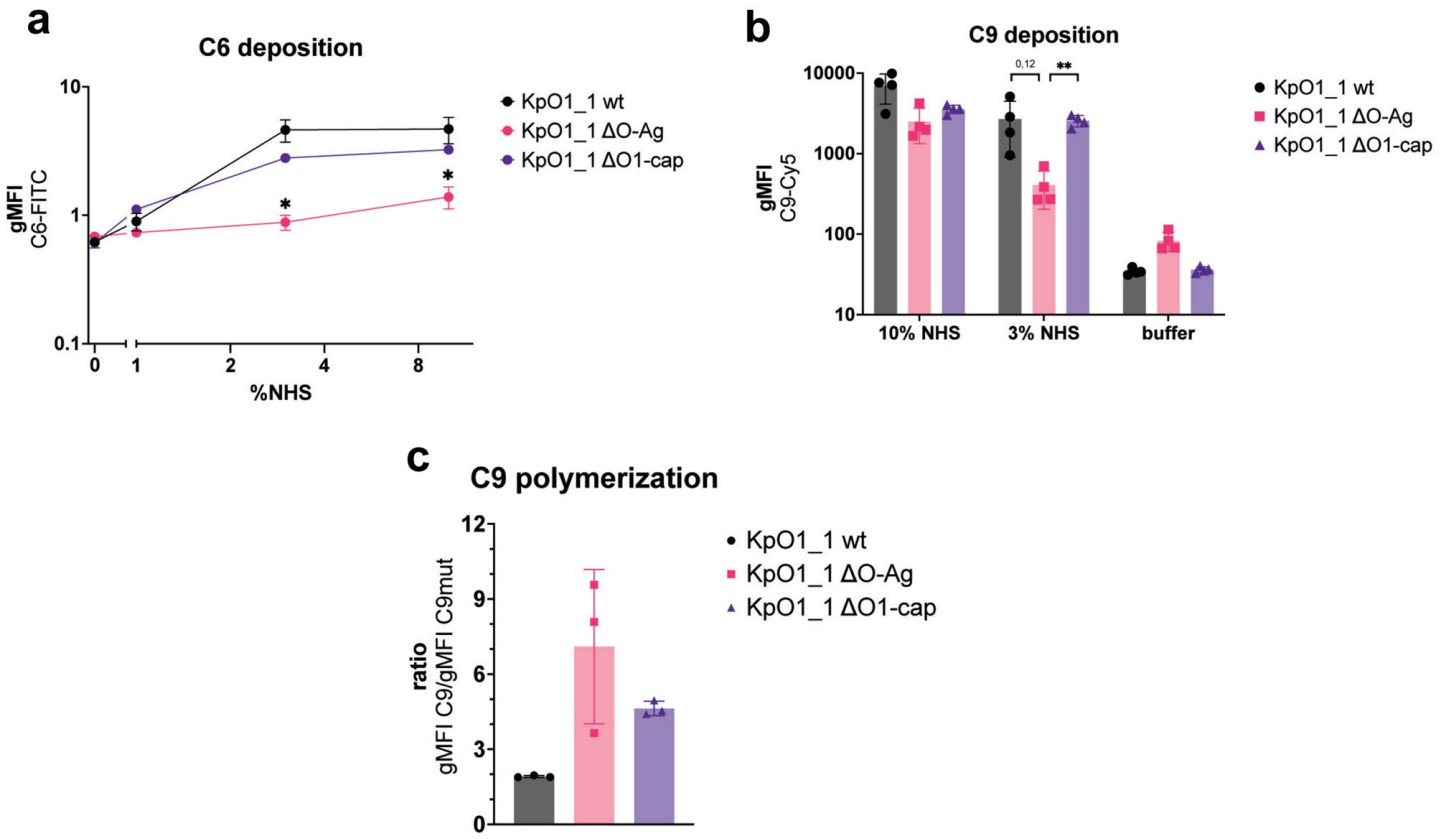
Presence of O-antigen correlates with increased deposition of terminal complement components. Bacteria were exposed for 15 min at 37 °C to either: (**a**) NHS with serological concentrations of directly labelled C6 added. C6 deposition was determined using flow cytometry and measuring geometric mean fluorescence intensity of directly labelled C6-FITC. (**b**) C9 depleted serum repleted with serological concentrations of directly labelled C9-Cy5. C9 deposition was determined using flow cytometry and measuring geometric mean fluorescence intensity of directly labelled C9-Cy5. Bacteria used were transformed to express RFP. (**c**) Ratios shown were calculated by dividing the geometric mean fluorescence values of bacteria with directly labelled C9wt by geometric mean fluorescence values of bacteria with C9_TMH1-lock_. Data shown in (**a**), (**b**) and (**c**) represent mean values ± SD of at least three independent experiments. Statistical analysis in (**a**) was done using paired t-test. Statistical analysis in (**b**) was done using a paired one-way ANOVA with Tukey’s multiple comparisons’ test. Significance shown as * p ≤ 0.05 or ** p ≤ 0.01.

Deletion of the entire O1-antigen or only the O1-cap enhances complement-mediated killing despite their difference in C5 convertase activity. This raised questions about the downstream MAC formation. After its formation, the C5 cleavage product C5b will bind C6 to form the C5b6 complex, followed by binding of proteins C7, C8, and multiple copies of C9 which will then form the MAC. To assess how the O1 antigen influences MAC assembly, we first analyzed C5b6 complex formation. For this, we spiked NHS with fluorescently labelled C6 and quantified the presence of fluorescently labelled C6 on the bacterial surface. Both wild-type and O1-cap deletion mutant deposited complement C6 to comparable levels (Fig. 4a). Upon deletion of the entire O1-antigen (ΔO-Ag)*,* levels of surface bound C6 were decreased, compared to wild-type. Altogether, the C6 deposition correlates with C5 conversion on the KpO1_1 wild-type and O1-antigen knockouts. We therefore hypothesize that the O1-antigen influences the last steps of MAC assembly. To this reason we determined deposition of C9, the final component of the MAC. For this, bacteria were exposed to serum depleted of C9, that was repleted with fluorescently labelled C9. In line with C6 deposition, the amount of C9 detected on the bacterial surface was the highest for KpO1_1 wild-type and O1 cap mutant, and lowest for the entire O1-antigen knockout (Figs. 4b, S8b). In conclusion, the amount of detectable terminal complement components on the bacterial surface does not explain the difference in killing caused by the O1-cap.

### O1-antigen prevents polymerization of C9 into functional MAC

Since the amount of C9 on the bacteria did not coincide with killing, we hypothesized that the O1-antigen interferes with polymerization and insertion of C9 into a functional MAC pore in the bacterial membrane. Together with the high oberserved C5 conversion, this would lead to increased deposition of MAC precursors, explaining the earlier results. In order to assess the capacity of C9 to bind to the bacterial membrane and polymerize, we used C8 depleted serum together with the previously described recombinant C9_TMH1-lock_ mutant. The C9_TMH1-lock_ mutant is unable to polymerize and form functional MAC pores^45^. Using C9_TMH1-lock_ should therefore result in the formation of MAC pre-pores containing only a single copy of C9. In contrast, repletion with wild-type C9 (C9wt) will result in polymerization and up to 18 copies of C9 will make up the MAC pore.

We pre-incubated bacteria with C8-depleted serum to allow for pre-pore formation on the bacteria. Next, bacteria were washed and incubated with purified C8 and either directly labelled C9 or C9_TMH1-lock_. For this, we used C9 and C9_TMH1-lock_ both labelled in a site-specific manner with the same fluorophore to a similar degree of labelling. We measured fluorescence of labelled C9wt and C9_TMH1-lock_ on bacteria (Fig. S9). Introduction of a washing step already led to decreased detectable levels of labelled C9 on KpO1_1 wild-type when compared to the setup in full serum (Figs. 4b, S9). This suggests that unstably inserted MAC precursors got released from the surface during the washing step. From these data, we calculated the ratio between the fluorescence values of C9wt and C9_TMH1-lock_. A high C9 : C9_TMH1-lock_ ratio of the complete O1-antigen knockout strain indicated that polymerization of C9 is taking place and C9 is forming a MAC pore (Fig. 4c). Conversely, the wild-type strain showed almost no difference in fluorescence levels between wild-type C9 and C9_TMH1-lock_, and therefore a very low C9 polymerization ratio, suggesting that C9 is unable to polymerize on its surface. Based on the decreased viability upon serum exposure, we expected the O1-antigen cap mutant to also show a high C9 : C9_TMH1-lock_ ratio. We observed an increased ratio compared to wild-type, however, lower compared to the full O1-antigen mutant. This indicates that part of the C9 can polymerize and form MAC pores in the O1-cap mutant, thereby killing the bacteria.

### O1-antigen prevents insertion of C9 into the bacterial membrane

**Fig. 5:**
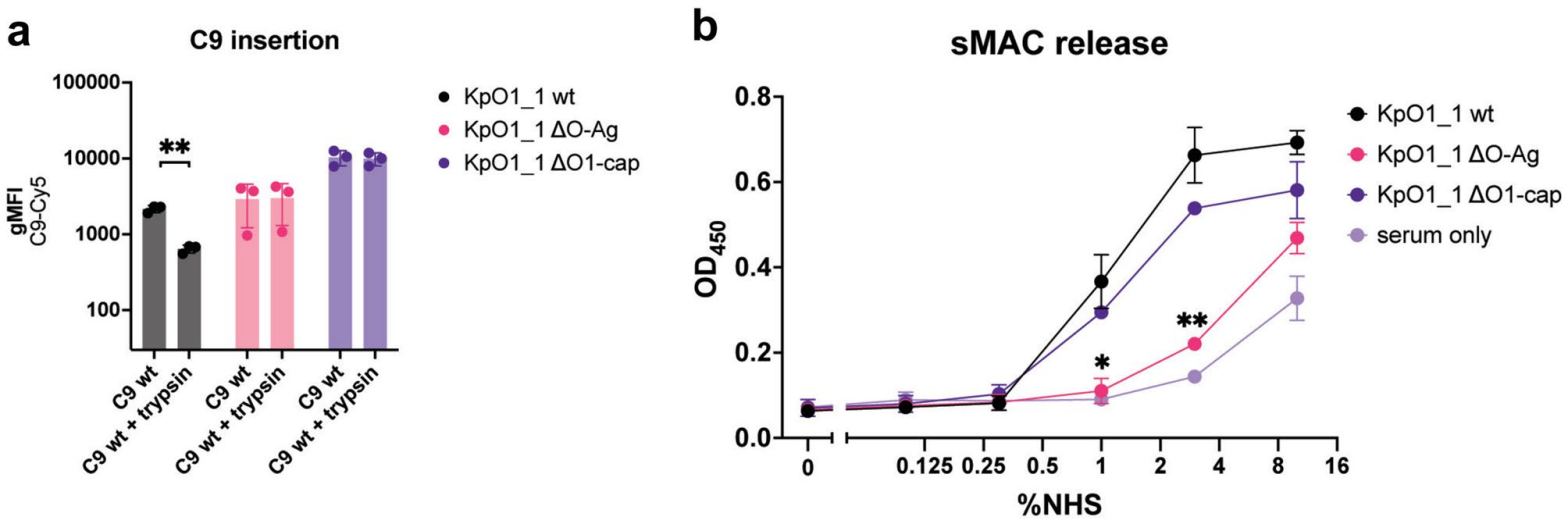
O-antigen prevents correct polymerization and insertion of C9 on the bacterial surface. (**a**) RFP-positive bacteria where incubated for 15 min at 37 °C in 10% C8 depleted serum to allow MAC precursor formation, washed, followed by incubation for 15 min at 37 °C with serological concentrations of C8 and directly labelled C9, followed by incubation for 15 min at 37 °C with 10 µg/ml chymotrypsin. MAC shave-off was determined through measuring geometric mean fluorescence intensity of labelled C9 using flow cytometry. (**b**) Bacteria where incubated for 15 min at 37 °C in C6 depleted serum repleted with serological concentrations of biotinylated C6. sMAC release was determined via sandwich ELISA using α-C9neo-antibody (clone aE11), and streptavidin. Data shown in all panels represent mean values ± SD of at least three independent experiments. Statistical analysis of a) and b) was done using paired t-test. Significance shown as *p ≤ 0.05 or **p ≤ 0.01.

Since polymerization of C9 was inhibited on KpO1_1, we wondered if C9 binds to the C5b-8 complex and insert into the bacterial outer membrane. For this, we used the same setup as for the C9 polymerization assay, but introducing an additional trypsinization step to digest and thereby remove incorrectly assembled MAC pores^6^. For the KpO1_1 wild-type strain, trypsinization led to a further decrease of detectable C9 on the bacterial surface, confirming that the O1-antigen prevents anchoring of MAC on its surface (Fig. 5a). In contrast, for both the full O1-antigen knockout, and O1-antigen cap knockout trypsinization did not lead to a decrease in detectable C9 on the bacterial surface. Taken together, this suggests that the O1-antigen has an influence on bacterial survival by hindering MAC insertion.

### O1-antigen leads to increased sMAC release

Since KpO1_1 wild-type showed reduced insertion of C9 into the bacterial membrane, this left us with the question whether the presence of the O1-antigen leads to more release of MAC pre-pores from the bacterial surface. As has been described for *E. coli*, presence of an O-antigen can interfere with attachment of MAC precursors on the bacterial outer membrane of Gram-negatives, leading to the release of pre-pores also known as soluble MAC (sMAC) into the supernatant ^21,46^.

We therefore determined the release of soluble MAC from the bacterial surface using a sandwich ELISA. We found an increase of sMAC formation in the supernatant of the O1 wild-type and O1-cap knockout (Fig. 5b). In contrast, in absence of entire O1-antigen, formation of sMAC was only slightly increased compared to spontaneous formation in serum. To summarize, our data suggest that *Klebsiella* O1-antigen prevents insertion and polymerization of C9 into a functional MAC pore, which is released as sMAC from the bacterial surface.

## Discussion

**Fig. 6:**
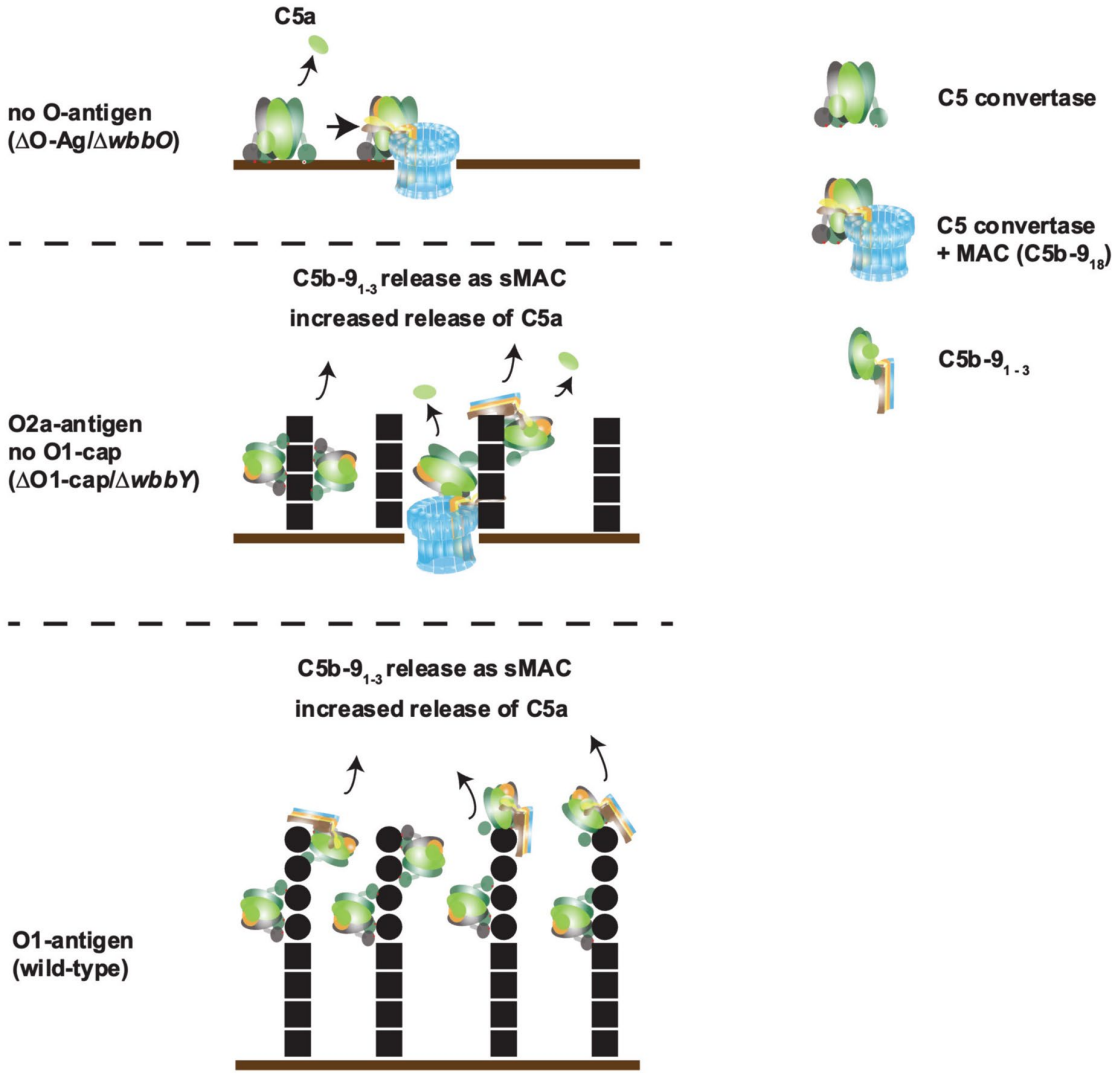
Schematic representation of MAC formation on LPS O1-antigen. If no O-antigen is present, C5 convertase is active and the MAC can form in the bacterial membrane and perforate it. When only O2a-antigen is present, C5a conversion is enhanced. The increased C5 conversion results in enhanced deposition of MAC precursors. Part of the MAC precursors are released as C5b9_1-3_ (sMAC) from the bacterial surface. However, some pores can still correctly form in the outer membrane and induce killing. In presence of O1-antigen, C9 polymerization is inhibited, preventing MAC formation and killing.

Insertion and correct polymerization of the membrane attack complex (MAC) into the outer membrane is paramount for killing of Gram-negatives such as *Klebsiella pneumoniae* by the complement system^6^. Although the link between expression of LPS O-antigen and resistance to MAC-mediated killing has been established for *Klebsiella*^25,29,47^, we here show that specifically the LPS O1-antigen of *Klebsiella* prevents polymerization of C9 into functional MAC, thereby preventing killing (Fig. 6).

Our findings confirm previous data showing that *Klebsiella pneumoniae* expressing an O1-antigen are more resistant to MAC-mediated killing than those expressing an O2a-antigen. By deletion of genes involved in O1-antigen synthesis, we could pinpoint MAC resistance to not only the presence of the O1-antigen, but we could also show that specifically the O1-cap (d-galactan II repeating units) is required to create a MAC-resistant phenotype. Next to the O-antigen, the capsule can contribute to bacterial survival in serum^40,48^. However, for strain KpO1_1, we were not able to measure an effect of the capsule on bacterial survival in serum. We only analyzed the capsule mutant of KpO1_1, and therefore the finding that the capsule does not play a role in complement resistance cannot be generalized to other strains. Since the O1 antigen showed a major phenotype in membrane damage and survival assays, we focused on the role of the O1-antigen.

Our results confirm the previously reported discrepancy in serum-resistance between the O1 and O2a serotypes, namely that O1-antigen strains are more resistant to MAC-mediated killing than O2-antigen strains^20,25,36^. While the O1-antigen and its structure have been known for more than thirty years^49^, the two genes responsible for the synthesis of d-galactan II, *wbbY* and *wbbZ*, respectively, have been only recently characterized^38,39^. While *wbbY* has been shown to be an essential glycosyltransferase for the synthesis of d-galactan II, the role of *wbbZ*, a pyruvyltransferase, remains understudied. Additionally, these two genes are located between transposable elements and might have gotten acquired via lateral gene transfer^35^.

Despite their susceptibility to serum, strains expressing an O2-antigen are commonly found among *Klebsiella* serotypes causing infections in the blood stream^24^. This could be due to the fact that the O2-antigen seems to be less likely recognized by antibodies due to shielding by capsule and therefore more resistant against recognition by antibodies and opsonophagocytic uptake^24,35^.

Our mechanistic studies show that expression of O1-antigen does not block early steps of complement activation. Instead, expression of the O-antigen leads to increased deposition of C3b and C5 conversion and therefore high levels of bacterium-bound C5b6 and even C5b-9 complexes. However, deposited C5b-9 complexes are not functional on O1-antigen strains, because the complexes are unable to correctly insert into the bacterial outer membrane and polymerize into a functional pore. Our finding that deposition of C3b was not impaired by the presence of an O-antigen, is in disagreement with previous reports, where serum-resistant *K. pneumoniae* LPS smooth strains (O1-strains) deposited less C3 than their serum-sensitive LPS rough (O-antigen negative strains)^47^. We can, however, confirm earlier findings that the O1-antigen does not lead to evasion of complement activation^19^. Surprisingly, in presence of O1-antigen, we observed higher levels of C3b deposition, which are also reflected in the higher amount of C5 converted to C5a and C5b. Presence of the shorter O2a-antigen (O1-cap deletion) still leads to high C5 conversion, while deletion of the entire O1-antigen leads to drastically lower C5 conversion rates. This is partly in line with previous findings, that serum-resistant Gram-negatives which express an LPS O-antigen can potently convert C5^21^. However, it seems the shorter O2-antigen is enough to trigger the increased C5 conversion, even though it does not lead to MAC-resistance. The strong release of pro-inflammatory C5a in response to LPS constitutes a great risk factor for an anaphylactic shock, as has been shown in mice^50^.

The potent C5 conversion further correlated with deposition of C9, the terminal protein of the MAC. Surprisingly, the amount of detectable C9 on the bacterial surface did not correlate with killing. This is seemingly in line with previous findings of potent MAC deposition on serum-resistant Gram-negative bacteria such as *Acinetobacter*, but also on Gram-positives like *Streptococcus* ^51,52^. However, it should be noted that the frequently used detection antibody for MAC, aE11, is unable to discriminate the C5b-9 pre-pore from a fully polymerized MAC pore, as it binds to two adjacent C9 molecules^53^. Therefore, researching if C9 is polymerizing turns out essential for mechanistic studies. By using directly labelled C9 and the recently described C9 variant that locks C9 in its monomeric form, we could show that expression of the O1-antigen prevents polymerization of C9 into a bactericidal pore. We can link this mechanism not only to MAC-resistant strains, but to the presence of the O1-antigen specifically. In presence of the full O1-antigen, detectable levels of C9 are highest, while C9 polymerization and insertion into the bacterial membrane are impaired. As a consequence, sMAC is released. While the shorter O2a-antigen might not be sufficient to prevent MAC-mediated killing, it nevertheless decreases the efficiency of how the MAC can form on the bacterial surface, whereas the O1-antigen prevents MAC-formation altogether. The underlying mechanism might be explained by the excessive consumption of complement components by C5 convertases resulting in MAC pre-pores. We speculate that the LPS O1-antigen, by activating more C5 convertases, changes the local ratio of C5 to C9 to conditions where only a suboptimal amount of C9 molecules are available per C5b6 pre-pore complex^21^. Unable to correctly anchor, insert into the membrane and to further polymerize, these MAC pre-pores would get released into the bacterial supernatant as soluble MAC. Doorduijn et al. have reported a similar finding in *E. coli*, where presence of an O-antigen prevented polymerization of C9 and lead to increased C5 conversion and sMAC generation ^21,22^.

In the case of a shorter O2-antigen, convertase activity will still be increased and lead to partial release of MAC precursors as sMAC. However, the O2a-antigen gives less protection against membrane insertion of the MAC, and some pores form correctly and penetrate the outer membrane, thereby killing the bacteria. These findings highlight the importance of the O1-antigen for MAC-resistance in *K. pneumoniae*. It might also serve as a possible explanation why O1-bearing *K. pneumoniae* are the most common serotype of clinical relevance^35^. Potentially, these data suggest a multi-faceted role of the O1-antigen during an infection. Not only does the O1-antigen render *K. pneumoniae* non-susceptible to MAC-killing, but the increased release of anaphylatoxin C5a also pose a high inflammatory burden during an ongoing infection. The release of soluble MAC components in both cases might serve as a biomarker of ongoing infections with *K. pneumoniae*.

In light of the rise of multidrug resistant hypervirulent *Klebsiella* strains^54^, understanding how *K. pneumoniae* resists exposure to the complement system and killing via the MAC is of increasing importance. This study gives molecular insights into how expression of the LPS O-antigen helps *K. pneumoniae* withstand direct killing through the MAC.

## Methods

### Serum, reagents and bacterial strains

Normal human serum (NHS) was derived by pooling serum of ~ 20 healthy donors and prepared as described before^6^. Heat-inactivated serum was obtained by incubating NHS at 56 °C for 30 min. OmCI was produced and purified as previously described^33^. Eculizumab was kindly provided by Frank Beurskens (Genmab, Utrecht, The Netherlands). RPMI (ThermoFisher) supplemented with 0.05% human serum albumin (HSA, Sanquin), further referred to as RPMI buffer, was used in all experiments, unless otherwise stated. Sera depleted of complement factors C6, C8 and C9 were obtained from Complement Technology. *Klebsiella pneumoniae* clinical isolates were collected during routine diagnostics in the medical microbiology department in the University Medical Centre Utrecht, The Netherlands, kindly provided by Jelle Scharringa, Janetta Top and Ad Fluit (see Table 1). Determination of capsule and O-antigen type of clinical *Klebsiella pneumoniae* isolates was performed using the Kaptive online tool (v. 2.0.4)^15,30,31^. C9 deposition and trypsin shaving experiments were performed using clinical isolates and knockout mutants transformed with plasmid pTU2-A-RFP (gift from Paul Freemont, Addgene plasmid #72954)^55^.

**Table 1:** Strains used in this study.

Strain	Description	K-type	O-type	Origin
O1_1 (UM-05-A#1)		KL114	O1	Verschuren et al*.*, unpublished
O1_1 Δ*wbbO*	O-antigen KO	KL114	–	van der Lans et al*.*, unpublished
O1_1 Δ*wbaP*	Capsule KO	–	O1	van der Lans et al*.*, unpublished
O1_1 Δ*wbbY*	O1-cap KO	KL114	O2a	van der Lans et al*.*, unpublished
O1_1 Δ*wbbO*::pTU2-*wbbO*	O-antigen KO, complemented with O-antigen	KL114	O1	This study
O1_1 Δ*wbbY*::pTU2-*wbbYZ*	O1-cap KO, complemented	KL114	O1	This study
O1_2 (UM-2A#3.1)		KL20	O1	Verschuren et al*.*, unpublished
O1_2 Δ*wbbO*	O-antigen KO	KL20	–	This study
O1_2 Δ*wbbY*	O1-cap KO	KL20	O2a	This study
O1_2 Δ*wbbY*::pTU2-*wbbYZ*	O1-cap KO, complemented	KL20	O1	This study
O1_3 (UM13B#1)		KL19	O1	Verschuren et al*.*, unpublished
O1_4 (MB-17667)		KL30-D1	O1	Van den Bunt et al*.* ^62^
O1_5 (MA-28030)		KL10	O1	Van den Bunt et al*.* ^62^
O2_1 (UM-05-B-#1)		KL114	O2a	Verschuren et al*.*, unpublished
O2_1 Δ*wbbO*	O-antigen KO	KL114	–	This study
O2_2 (MC-04166)		KL136	O2afg	Van den Bunt et al*.* ^62^
O2_3		KL102	O2afg	This study
O2_4 (JS367*)		KL9	O2afg	van der Lans et al*.*, unpublished
O2_5		KL107	O2afg	This study
O3_1 (MB-16059)		KL2	O3b	Van den Bunt et al*.* ^62^
O3_2 (MA-25932)		KL60	O3b	Van den Bunt et al*.* ^62^
O3_3 (MA-31364)		KL125	O3b	Van den Bunt et al*.* ^62^
O3_4 (MC-40993)		No confidence match	O3b	Van den Bunt et al*.* ^62^
O3_5 (MA-47281)		No confidence match	O3b	Van den Bunt et al*.* ^62^
O4_1 (MA-02666)		KL151	O4	Van den Bunt et al*.* ^62^
O4_2 (MA-49950)		KL17	O4	Van den Bunt et al*.* ^62^
O4_3		KL36	O4	This study
O5_1 (MC-39002)		No confidence match	O5	Van den Bunt et al*.* ^62^
O5_2 (MA-44575)		KL10	O5	Van den Bunt et al*.* ^62^
O104_1 (MA-18898)		KL14	OL104	Van den Bunt et al*.* ^62^
Ox_1 (MA-18684)		No confidence match	no confidence match	Van den Bunt et al*.* ^62^
Ox_2 (MA-48971)		No confidence match	no confidence match	Van den Bunt et al*.* ^62^

### Site-specific labelling of MAC components

To produce fluorescently labelled C9, C9-3xGGGGS-LPETG-6xHis was recombinantly expressed in Expi293F cells and site-specifically labelled with Cy5 via C-terminal sortagging as done previously^56^. C9_TMH1-lock_ was produced and labelled in the same manner, as described previously^21^. Biotinylated C6 and FITC-labelled C6 were produced in a similar manner, by expressing and isolating C6-LPETGG-6xHis, as previously described^7^ and subsequent C-terminal sortagging with GGGK-biotin (kindly provided by Louris Feitsma, Department of Crystal and Structural Chemistry, Bijvoet Institute) or GGGK-FITC. C6 and C9 were labelled with fluorescent probes as described previously. 50 μM of protein with C-terminal LPETGG-His tag was incubated with 25 μM His-tagged sortase-A7+ (recombinantly expressed in *E. coli*) and 1 mM GGG-substrate in Tris/NaCl buffer (50 mM Tris/300 mM NaCl at pH 7.8) for two hours at 4 °C. GGGK-FITC (Isogen Life Science) was used for C6-LPETGG-His and GGGK-azide (Genscript) for C9-LPETGG-His. Sortagged proteins were purified on a HisTrap FF column (GE Healthcare), which captures protein that was not sortagged and still contains a His-tag. In addition, FITC-labelled C6 was purified by SEC on a Superdex 200 Increase column on the Akta Explorer with PBS ^22^. GGG-azide labelled proteins were concentrated to 25 μM on a 30 kDa Amicon Tube (Merck Millipore) in Tris/NaCl buffer and next labelled with 100 μM DBCO-Cy5 (Sigma Aldrich) via copper-free click chemistry for 3h at 4 °C. Finally, Cy5-labelled proteins were also purified by SEC on a Superdex 200 Increase column with PBS. Labelling of the proteins was monitored during SEC by measuring absorbance at 280 nm (protein), 488 nm (FITC) and 633 nm (Cy5) nm and finally verified by SDS-PAGE by measuring in-gel fluorescence with LAS4000 Imagequant (GE Healthcare)^21^.

### Bacterial culture

Bacteria used in this study are shown in Table 1. Bacteria were freshly plated from glycerol stocks on blood agar plates. Single colonies were picked and cultured overnight at 37 °C in liquid LB medium under shaking conditions. The following day, bacteria were diluted 100-fold in fresh LB medium and incubated at 37 °C shaking until an OD_600_ of ~ 0.5 was reached. Bacteria were then washed with RPMI buffer and resuspended to a final OD_600_ of 1 for experiments.

### SYTOX™ assay

Bacteria (final OD600 = 0.05; ~ 5 × 10^7^ bacteria/ml) were incubated with 1 µM SYTOX™ Green nucleic acid stain (Invitrogen) and serum/heat-inactivated serum/serum with C5 inhibitors for 2 h at 37 °C in a Clariostar plate reader (BMG labtech). C5 inhibition was achieved by adding 20 µg/ml of Eculizumab and 20 µg/ml OmCI. Sytox Green fluorescence was measured using an excitation wavelength of 484–15 nm and an emission wavelength of 527–20 every 90 s.

### Bactericidal assays (CFU)

After the Sytox assay, bacterial cultures were pre-diluted 1000-fold in PBS. tenfold serial dilutions of samples were prepared in PBS and spotted in duplicate on LB agar plates, followed by overnight incubation at 37 °C. Colony forming units count was determined the next day and the correspondent concentration of CFU/ml was calculated. Survival was calculated by dividing the sample’s CFU/ml by the CFU/ml at t = 0.

#### RFP-labelling

Bacterial subculture (OD_600_ ~ 0.5) was spun down at 4 °C and washed three times with ice-cold 10% (v/v) Glycerol. Approximately 100 ng of pTU2-A-RFP plasmid was added to 50 µl competent bacteria, followed by electroporation on a Bio-Rad Gene Pulser Xcell (1.8 kV, 25 µF, 200 Ω). After electroporation, 250 µl of super optimal conditions (SOC, Invitrogen) medium was added, and bacteria were incubated at 37 °C for 90 min, before plating on selective LB agar plates with 20 µg/ml chloramphenicol and overnight incubation at 37 °C. Plasmid pTU2-A-RFP was a gift from Paul Freemont (Addgene plasmid #72954)^55^.

### Single gene deletions and complementations

Competent bacteria were transformed with 100 ng temperature-sensitive pREDKI helper plasmid^57^ as described above, but cultured at 30 °C and in presence of 50 µg/ml kanamycin as selective marker. Plasmid-bearing bacteria were cultured overnight in LB with kanamycin at 30 °C while shaking. The subculture was incubated with the addition of L-Arabinose (15 mM final concentration) to induce the λ-red recombination system, and then made competent again using the aforementioned method. Competent bacteria were then transformed by electroporation with 100 ng of linear DNA fragments consisting of a gentamycin resistance cassette flanked by 50 bp overhang sequences homolog to the flanking sequences of the gene to be knocked out. Primer sequences for the primers used for the generation of linear DNA fragments can be found in Table 2. Bacteria were incubated at 30 °C for 90 min in SOC, before plating on selective LB agar plates with 50 µg/ml gentamycin and incubation overnight at 30 °C. Successful knock-out colonies were confirmed by PCR, and confirmed clones were cultured overnight at 37 °C in LB + 50 µg/ml gentamycin to lose the helper plasmid. For complementations, genes were cloned onto plasmid pTU2 under regulation of the corresponding promotor of strain KpO1_1. Plasmids were then transformed into bacteria made competent using the method mentioned above.

**Table 2:** Primers used in this study.

Primer name	Sequence 5ʹ–3ʹ
WbbO_For	CACATGGCATAAAAGGTATTAGTGGTAAGTATCATTAAGTGGGCATAGCACTAGTGAGCTCATGCATGATCG
WbbO_Rev	GGTAAAGAAACAATATCTGGCAATTAATAAAAAAGGATAACCGTTTGATCGACAACGAATTGGGGATCTTGAAGTACC
WbbY_For	CACTACTTCAATTCACTAATATCATAGAAAAGTCTAGGTTACAAAGGAAGGGTTACACTAGTGAGCTCATGCATGATCG
WbbYZ_Rev	TAGCTGAAAGTTAATATTATTTTTGCGGAGCCCTTTCGGGCCCCGAATATTACTCGAATTGGGGATCTTGAAGTACC
WbaP_For	ATGACGCATTTTACTAAAAATGTATTGTGCAGTGTATTTTTAGCTACAGCAGCTAGTGAGCTCATGCATGATCG
WbaP_Rev	TTAGTATGCGCCATCTCTTTTTAAAACGACACCAACCGTTTTAAACAAAATAGCGAATTGGGGATCTTGAAGTACC
WbaP_Check_For	ACGATTCGAACAAAGTGGTGT
WbaP_Check_Rev	TGCCCATGGGAATATCCTGT
KO_cass_DN_For	TAAATTGTCACAACGCCGCG
KO_cass_UP_Rev	CGTTCGGTCAAGGTTCTGGA

#### Antibody deposition assays

For deposition of natural IgG and IgM from serum pool, bacteria (~ OD_600_ 0.05 final conc.) were incubated with NHS for 20 min at 4 °C shaking. Bacteria were then washed twice with RPMI buffer, followed by incubation with directly labelled goat-anti-human-AF405 (Invitrogen, A48275) or goat-anti-human-IgM-FITC (Southern Biotech, 2020-02) for 20 min at 4 °C shaking. Before FACS analysis, samples were diluted 1:10 in ice-cold 1% PFA in RPMI buffer. The *S. aureus* Newman strain used as positive control, is a *spa* (staphyolococcal protein A) and *sbi* (staphylococcal immunoglobulin-binding protein) double knockout mutant to not interfere with deposition of immunoglobulins on the surface (obtained from^58^).

For deposition of O-antigen specific monoclonal antibodies, bacteria (~ OD_600_ 0.05 final conc.) were first incubated with human monoclonal antibodies against *Klebsiella* LPS O1-antigen (sequence patented in^59^, produced in-house as described in^60^) for 30 min at 4 °C shaking. Bacteria were then washed with RPMI buffer, followed by incubation with directly labelled goat-anti-human-IgG-AF488 (2040-3, Southern Biotech) for 30 min at 4 °C shaking. Before FACS analysis, bacterial samples were diluted tenfold in RPMI buffer with 1% PFA.

#### C3b deposition

Bacterial culture (~ OD_600_ 0.05, appox. 5 × 10^7^ bacteria/ml final conc.) was incubated with serial dilutions of normal human serum (NHS) which was supplemented with C5-inhibitors Eculizumab (15 µg/ml) and OmCI (6 µg/ml) at 10% serum in a microtiter plate for 30 min at 37 °C shaking. Bacteria were then washed twice in RPMI-HSA, and resuspended with directly labelled anti-C3b antibody (bH6^61^, produced in-house^6^, 3 µg/ml final concentration in RPMI buffer). Bacteria were then washed once in RPMI-HSA and diluted to 2.5 × 10^6^ bacteria/ml in ice-cold RPMI buffer + 1% PFA before FACS analysis.

#### C6 deposition

Bacterial culture was incubated with serial dilutions of NHS together with directly labelled complement C6-FITC added in serological concentrations. Normal concentrations of MAC components in 100% serum are: 500 nM C6, 367 nM C8, and 845 nM C9. Incubation in a microtiter plate was 15 min at 37 °C while shaking. Incubation was stopped by diluting the sample 1:1 in ice-cold RPMI buffer + 1% PFA before FACS analysis.

#### C9 deposition

Bacterial culture was incubated with C9 depleted human serum complemented with serological concentrations of either directly labelled C9-Cy5 or C9_TMH1-lock_-Cy5 for 15 min at 37 °C shaking. Incubation was stopped by diluting the sample 1:10 in ice-cold RPMI buffer + 1% PFA before FACS analysis. C9 polymerization ratio was calculated by dividing the gMFI of C9-Cy5 by the gMFI of C9_TMH1-lock_-Cy5.

#### C9 polymerization and trypsin shaving

Bacteria were incubated with 10% C8 depleted serum for 15 min at 37 °C shaking, and then resuspended in RPMI buffer. Bacteria were then incubated with 10% serum equivalent serological concentrations of purified human C8 (Complement Technology), and either directly labelled C9-Cy5 or C9_TMH1-lock_-Cy5 for 15 min at 37 °C while shaking. After incubation with final complement components, bacteria were further incubated with either addition of 10 µg/ml chymotrypsin or RPMI buffer for 15 min at 37 °C while shaking. The reaction was stopped by diluting the samples 1:10 in ice-cold 1% PFA in RPMI buffer before FACS analysis.

#### Complement product activation ELISA’s

Bacteria (final conc. ~ OD_600_ 0.05) were incubated with serum titrations for 15 min at 37 °C shaking. For C5a, this was NHS, whereas for soluble MAC (sMAC) this was C6 depleted serum repleted with serological concentrations of biotinylated C6. Supernatants were then stored at − 20 °C until use. Serum supernatants were diluted 1:1 (v/v) in PBS-T supplemented with 1% BSA for sample analysis.

Nunc Maxisorp ELISA plates were coated with 1 µg/ml capture antibody in PBS at 4 °C overnight. For C5a, a commercially available kit was used, using C5a capture and detection antibodies specific for C5a and not native C5 (R&D, DY2037). For sMAC, capture was performed using mouse-α-C9neo-antibody (clone aE11, kindly provided by T. Mollnes and P. Garred). Blocking was performed by incubating with 4% BSA in PBS-T for one hour at 37 °C. Samples were then loaded and incubated for 1 h at room temperature. Plates were then incubated with detection antibodies for 1 h at RT. For sMAC, this was executed using 1:5000 HRP-conjugated Streptavidin (Southern Biotech). Washing between steps was performed three times with PBS-Tween 0.05% (PBS-T). Plates were developed for 2 min with fresh tetramethylbenzidine (TMB) substrate solution. Reaction was stopped using 2N sulfuric acid, and OD450 was then measured.

### Western blotting

Bacteria (~ OD_600_ 1 or 0.1, respectively) were incubated (1:1, v/v) with NHS at indicated concentrations for 15 min at 37 °C shaking. After washing, bacterial pellets were resuspended in PBS and diluted 1:1 in reducing SDS sample buffer supplemented with 50 µg/ml dithiotreitol (DTT) and incubated at 95 °C for 5 min. Samples were run on a 4–12% Bis–Tris gradient gel (Invitrogen) for 45 min at 200V in MES buffer. As controls, 10 µg of purified C5 or C9 were loaded. Proteins were transferred with the Trans-Blot Turbo transfer system (Bio-Rad) to 0.2 µM PVDF membranes (Bio-Rad). Initial blocking was performed using 4% skim milk (ELK, Campina) in PBS-T (PBS with 0.05% Tween) for 45 min at 37 °C. Primary staining was performed using a 1:500 dilution of either goat-anti-human-C5 (Complement Technology) or goat-anti-human-C9 (Complement Technology) in PBS-T supplemented with 1% ELK for 45 min at 37 °C. Secondary staining was performed using a 1:5000 dilution of HRP-conjugated pooled donkey antisera against goat IgG (H + L) (Southern Biotech) in PBS-T supplemented with 1% ELK for 45 min at 37 °C. In between all steps and after final staining washing was performed thrice using PBS-T. Finally membranes were developed with Pierce ECL Western Blotting Substrate (Thermo Fisher Scientific) for 1 min at room temperature and imaged on the LAS4000 Imagequant (GE Healthcare).

### FACS and data analysis

For deposition of C3b, C6, and binding of O-antigen specific monoclonal antibodies, bacterial samples were analyzed in a MACSquant VYB flow cytometer (Miltenyi) for forward scatter (FSC), side scatter (SSC), RFP, and AF488. For deposition of C9, and MAC shave-off, bacterial samples were analyzed on a BD FACSVerse flow cytometer for FSC, SSC, and Cy5. Flow cytometry data was analyzed in FlowJo version 10. Bacteria were gated on FSC and SSC.

## Supplementary Information

Supplementary Figures.

## Data Availability

The datasets generated during and/or analysed during the current study are available from the corresponding author on reasonable request. Unless otherwise stated, data collected as three independent biological replicates and analyzed using GraphPad Prism Version 10.1.1 (270).
