# Supplementary material for: Klebsiella LPS O1-antigen prevents complement-mediated killing by inhibiting C9 polymerization
Source: Sci Rep. 2024 Sep 5;14:20701. doi: 10.1038/s41598-024-71487-z (PMC11377433; doi:10.1038/s41598-024-71487-z)
Supplement: Supplementary file 1 — Supplementary Figures. [file 41598_2024_71487_MOESM1_ESM.pdf]

Klebsiella LPS O1-antigen prevents complement-mediated killing by inhibiting C9 polymerization

Frerich M Masson[1], Salvör Káradóttir[1], Sjors PA van der Lans[1], Dennis J Doorduijn[1], Carla JC de Haas[1], Suzan HM Rooijakkers[1], Bart W Bardoel[1*]

[1]Medical Microbiology, University Medical Centre Utrecht, Utrecht, The Netherlands
[*]corresponding author, B.W.Bardoel-2@umcutrecht.nl

Supplementary information

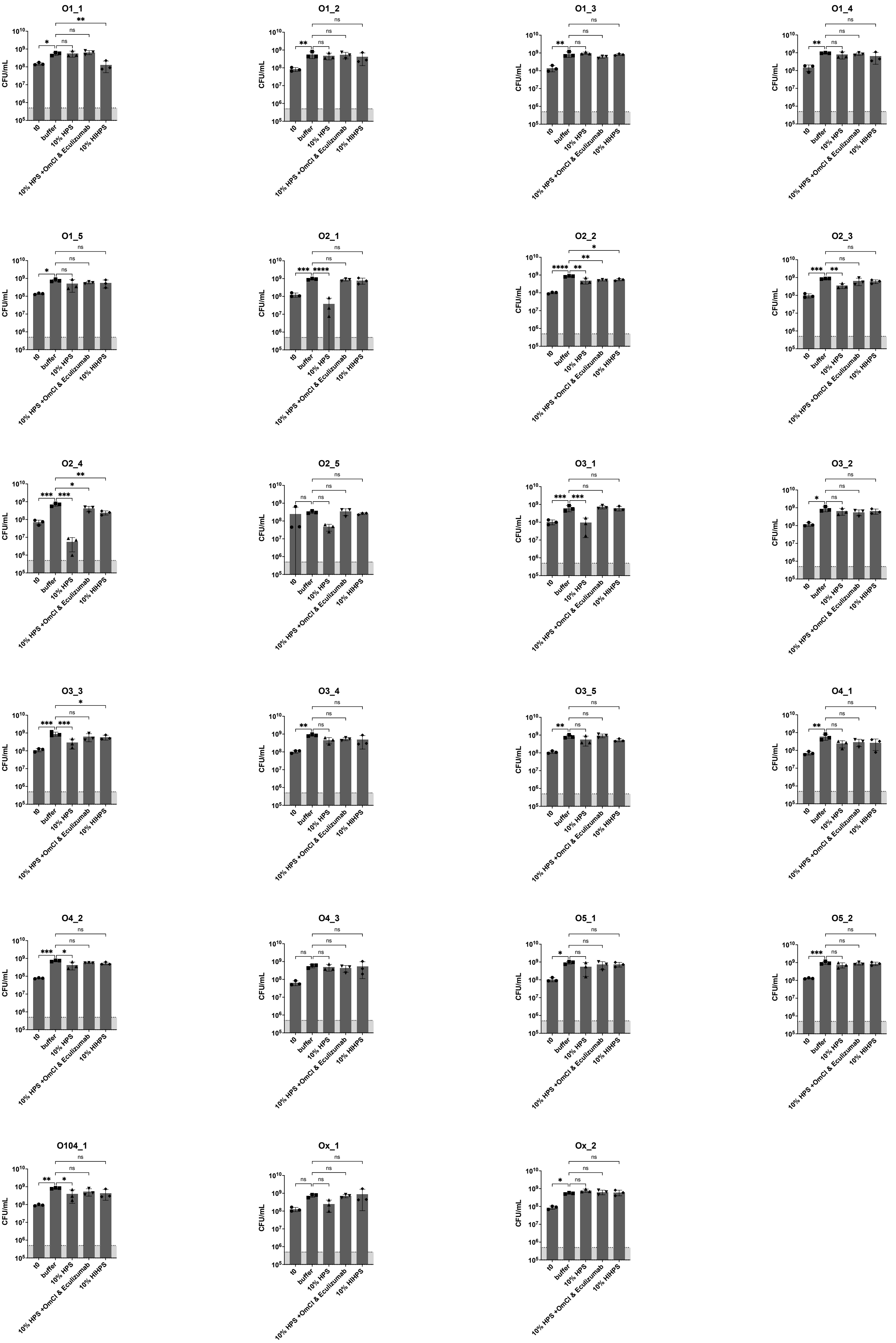

Supplementary Figure 1. Serum killing of clinical *K. pneumoniae* isolates is MAC-dependent.
Bacteria were exposed to 10% NHS, 10% HI NHS or 10% NHS with C5 inhibition at 37°C for 120 minutes. After serum exposure, serial dilutions of bacteria in PBS were plated and CFUs were assessed the next day. The grey bar is indicating the detection limit of the assay. Data shown represent mean values ± SD of three independent experiments. Statistical analysis was done using a paired one-way ANOVA with Tukey's multiple comparisons' test. Significance shown as * $p \leq 0.05$, ** $p \leq 0.01$, *** $p \leq 0.001$ or **** $p \leq 0.0001$.

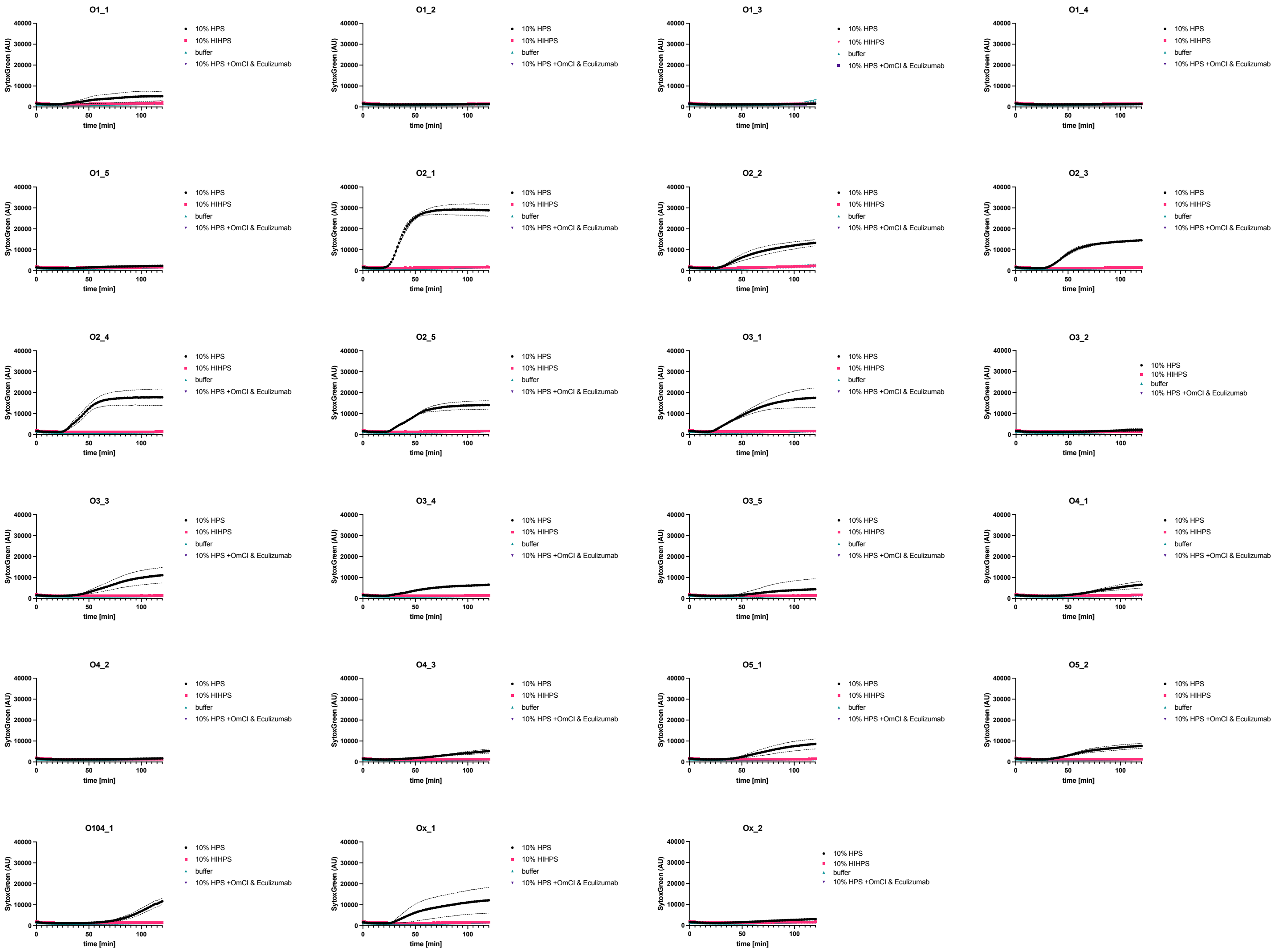

Supplementary Figure 2. Sytox Green signal correlates with serum killing of *K. pneumoniae* isolates. Bacteria were exposed to 10% NHS, 10% HI NHS or 10% NHS with C5 inhibition at 37°C for 120 minutes. Inner membrane damage was assessed using SytoxGreen DNA stain in a multiplate reader assay. Data shown represent mean values ± SD (thin lines) of three independent experiments.

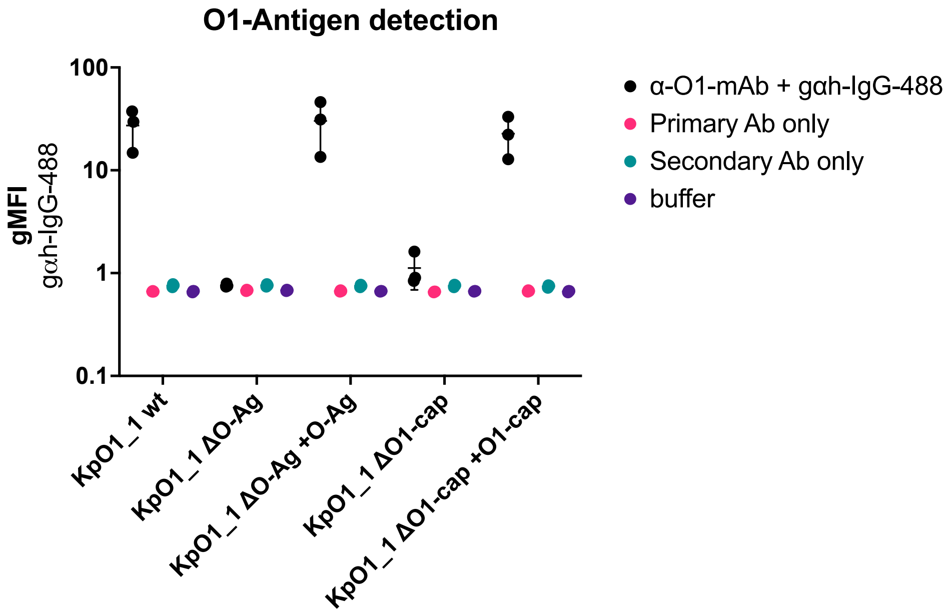

Supplementary Figure 3. Validation of O-antigen knockouts and complementations.
Bacteria were typed for presence of O1-antigen with α-O1-antigen antibody and fluorescent goat-α-human-IgG antibody. Presence of O1-antigen was plotted as gMFI of secondary antibody in the bacterial population. Data points shown represent mean values ± SD of three independent experiments.

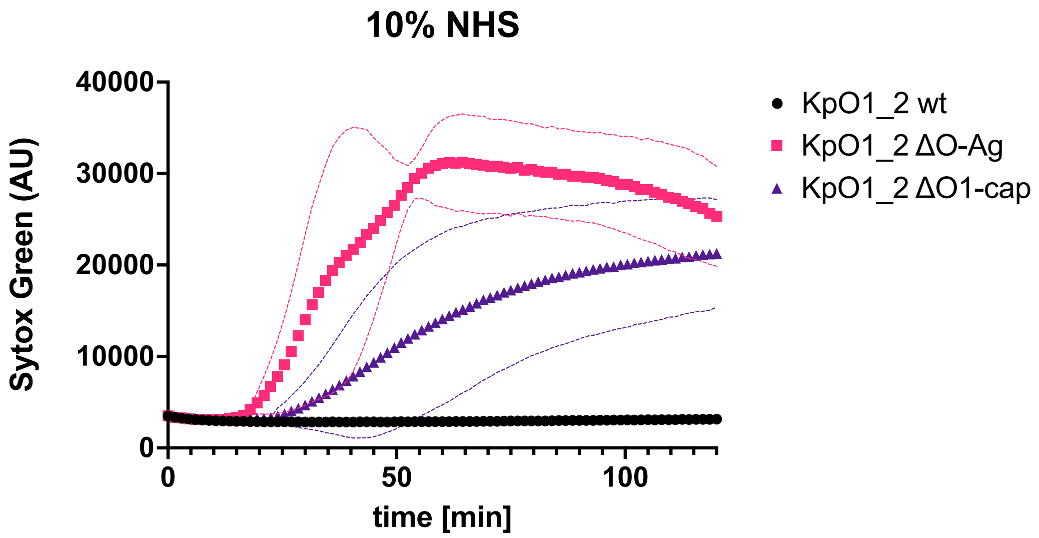

Supplementary figure 4 – Deletion of O-antigen or O1-cap leads to Sytox influx in another O1-strain
SytoxGreen fluorescence values of KpO1_2 wild-type and mutants after exposure to 10% NHS over time. Fluorescence was measured every 90 seconds. Data shown represent mean values ± SD (thin lines) of three independent experiments.

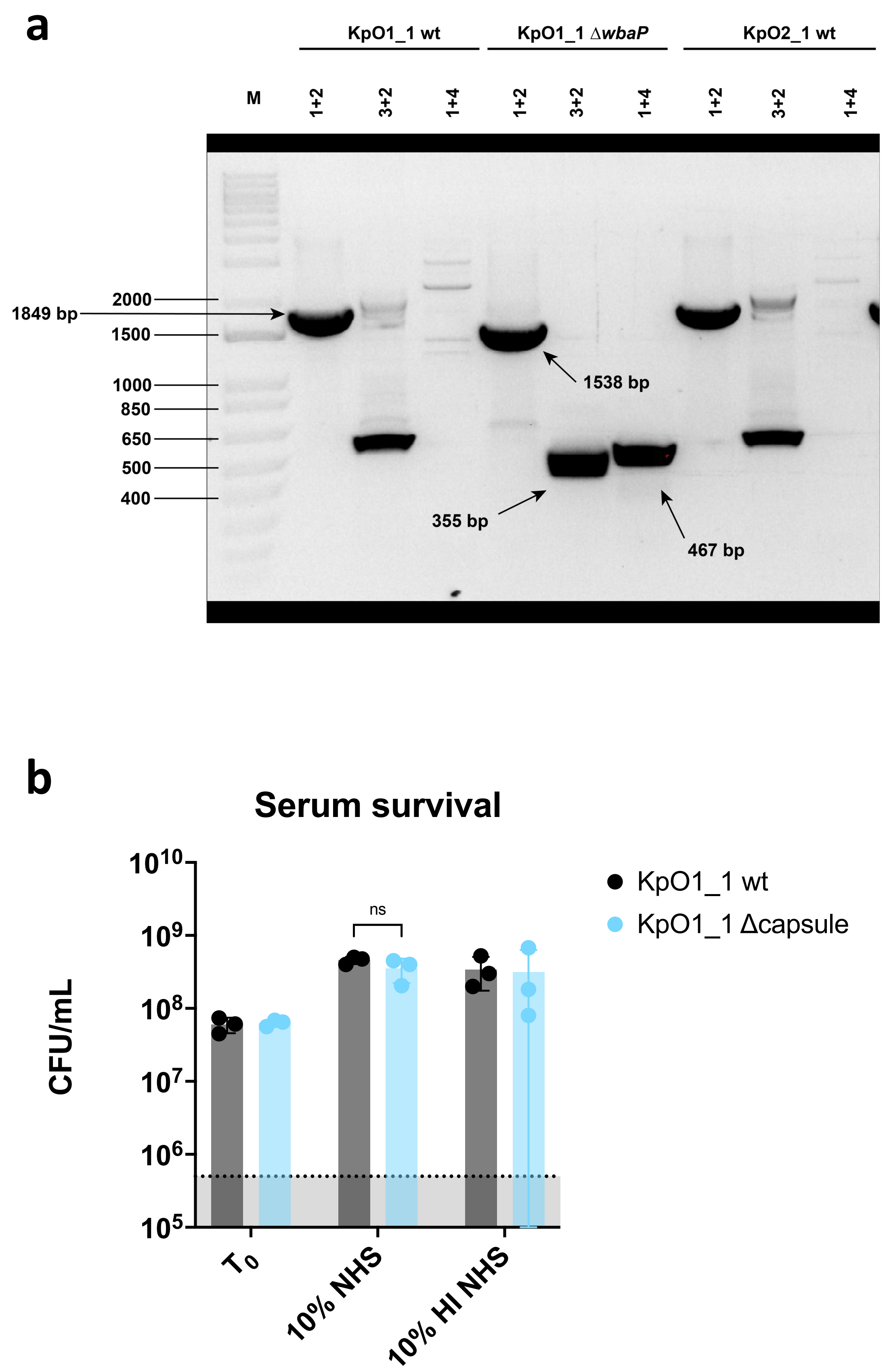

Supplementary figure 5 – Deletion of capsule does not influence bacterial survival after serum exposure. **a)** Vaildation of the capsule knockout in strain KpO1_1. Expected DNA fragment size for gene *wbaP* is 1849 bp in KpO1_1 and KpO2_1 wild-types and 1538 bp in deletion strain (as inidicated by arrows). Additional PCR on the inserted cassette and flanking gene region in the *wbaP* knockout should result in two DNA fragments of 355 bp and 467 bp size, respectively. Primers used: 1: wbaP_check_For, 2: wbaP_check_Rev, 3: KO_cass_DN_For, 4: KO_cass_UP_Rev **b)** CFU counts for both wild-type KpO1_1 and capsule deletion mutant after exposure to NHS or HI NHS at 37°C for 120 minutes. Data shown represent mean values ± SD of three independent experiments. Statistical analysis was done using a paired t-test. Significance is shown as ns, non-significant.

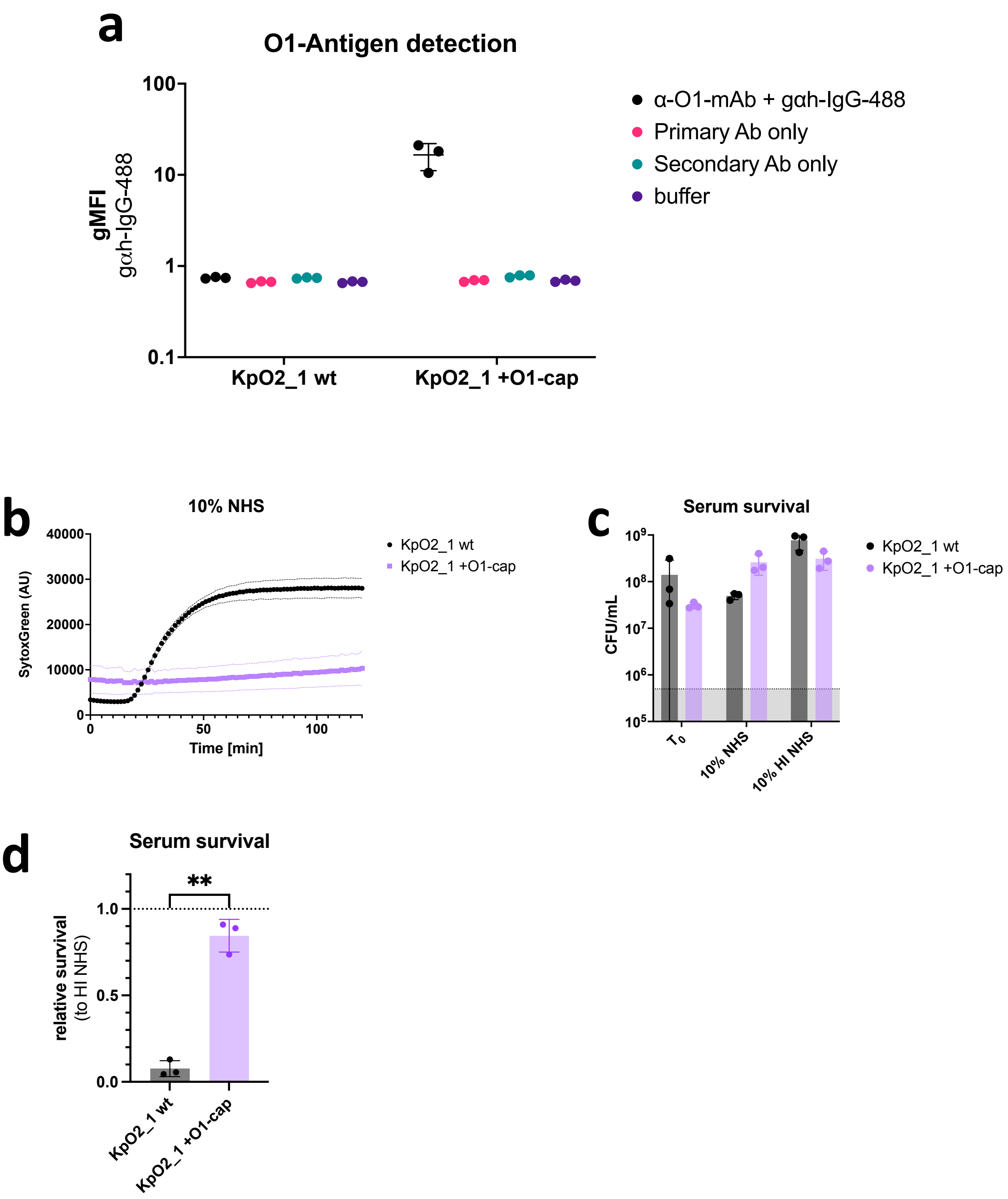

Supplementary figure 6 – Seroconversion with O1-cap renders serum sensitive strain resistant.

a) Bacteria were typed for presence of O1-antigen with α-O1-antigen antibody and fluorescent goat-α-human-IgG antibody. Presence of O1-antigen was plotted as gMFI of secondary antibody in the bacterial population. Data points shown represent mean values ± SD of three independent experiments. **b)** SytoxGreen fluorescence values of KpO2_1 and KpO2_1 complemented with O1-cap after exposure to 10% NHS over time. Fluorescence was measured every 90 seconds. **c)** CFU counts for both wild-type KpO2_1 and complemented mutant after exposure to either NHS or HI NHS at 37°C for 120 minutes. **d)** Relative survival of KpO2_1 wt and KpO2_1 cap-complemented to HI NHS buffer control. Dotted line indicates survival in HI NHS. Data shown in all panels represent mean values ± SD of three independent experiments. Statistical analysis was performed using a paired t-test. Significance shown as ** p ≤ 0.01.

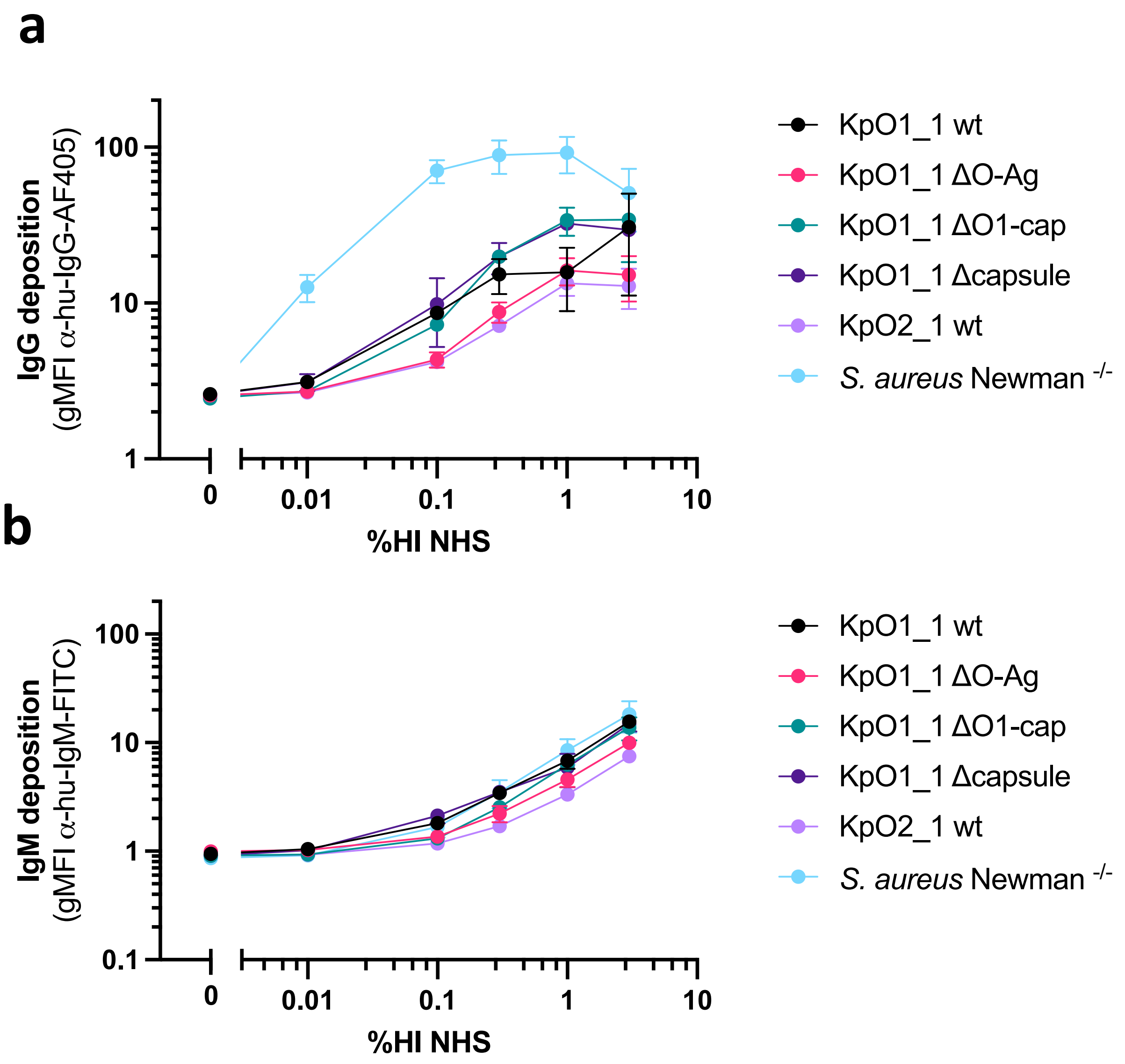

Supplementary Figure 7. Deposition of natural IgG and IgM from serum pool.
Bacteria were incubated in heat-inactivated (HI) normal human serum at 4 °C for 20 minutes. After washing, bacteria were incubated at 4 °C for 20 minutes with fluorescently anti-human-IgG-AF405 (a) or anti-human-IgM-FITC (b). **a)** IgG deposition from serum pool on bacteria. **b)** IgM deposition from serum pool on bacteria. Data points shown in a) & b) represent mean values ± SD of three independent experiments.

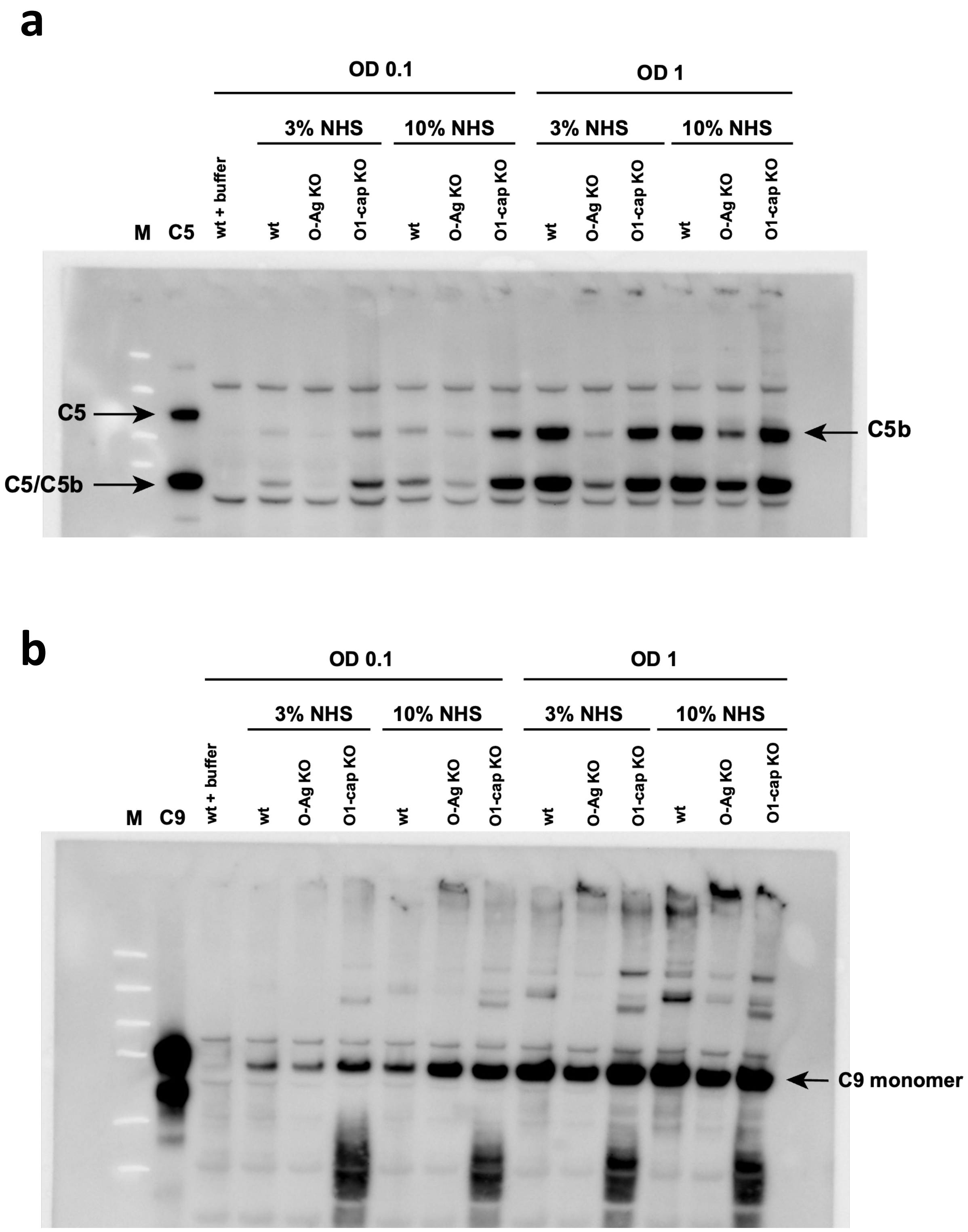

Supplementary Figure 8. O-antigen increases C5 conversion and C9 deposition.
Bacteria were incubated with normal human serum at 37 °C for 15 minutes. After washing, bacteria were run on a tris gradient gel with either 10 µg of C5 or 10 µg C9 as a control. Western blotting was performed for 45 minutes, followed by incubation with primary for 45 minutes at 37 °C using goat-anti-C5 or goat-anti-C9 primary antibodies, followed by donkey-anti-goat-HRP secondary antibody incubation for 45 minutes at 37 °C. Blot was incubated with detection reagents for 1 minute and analyzed on a LASimager. Data shown in a) & b) are representative images of two independent experiments.

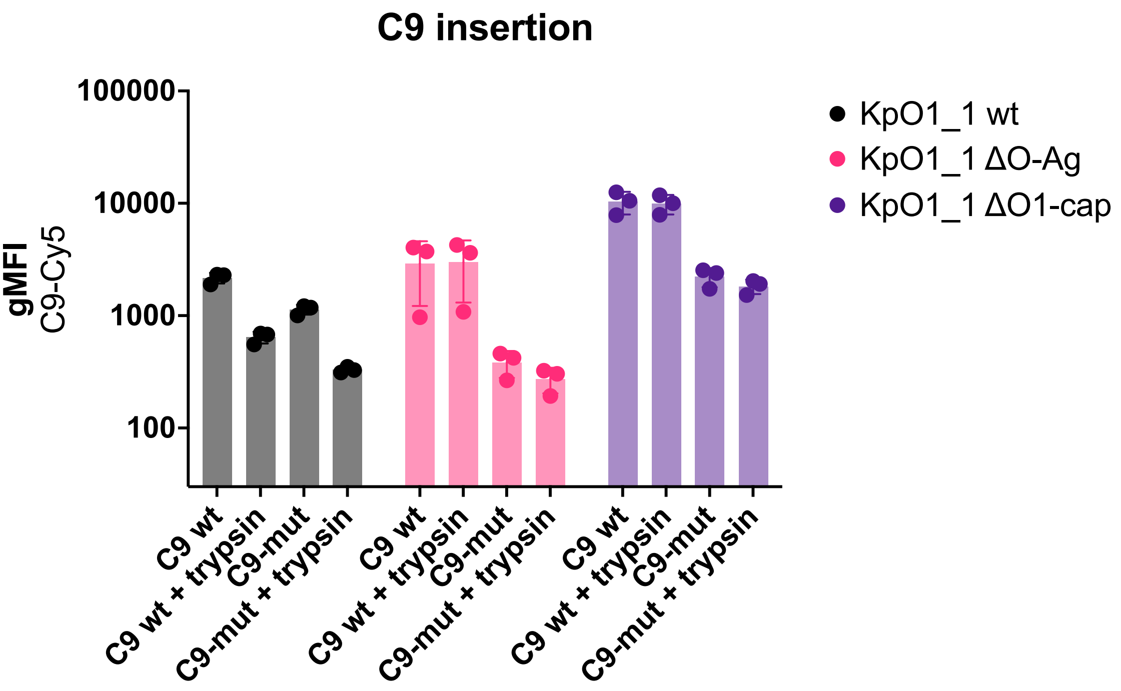

Supplementary figure 9. O1-antigen hinders C9 insertion into the bacterial membrane.
RFP-positive bacteria where incubated for 15 minutes at 37°C in 10% C8 depleted serum to allow MAC precursor formation, washed, followed by incubation for 15 minutes at 37°C with serological concentrations of C8 and directly labelled C9, followed by incubation for 15 minutes at 37°C with 10 µg/ml chymotrypsin. MAC shave-off was determined through measuring geometric mean fluorescence intensity of labelled C9 using flow cytometry. Data shown represent mean values ± SD of at least three independent experiments. Represented values were used to calculate the ratios of figure 4c.
